# Intensive care of a patient undergoing combined multi-organ cluster (“larynx–trachea–thyroid–hypopharynx–esophagus”) transplantation: A case report

**DOI:** 10.1097/MD.0000000000039081

**Published:** 2024-08-02

**Authors:** Jia Shen, Huan Liu, Yaodan Zhang, Yu Xu, Aiping Du, Yongming Tian

**Affiliations:** a Department of Critical Care Medicine, West China Hospital/West China School of Nursing, Sichuan University, Chengdu, China.

**Keywords:** cancer, combined transplantation, intensive care, multiple organ, transplantation

## Abstract

**Objective:**

The aim of this study was to summarize the intensive care experience of a patient undergoing combined multi-organ cluster (“larynx–trachea–thyroid–hypopharynx–esophagus”) transplantation.

**Methods:**

The intensive care management plan for this case was developed by a multidisciplinary team, with focus on 6 aspects: (1) stabilizing the circulation and reducing anastomotic tension by position management to improve the survival chances of transplanted organs, (2) adopting goal-directed analgesia and sedation protocols, as well as preventing anastomotic fistula, (3) implementing a bedside ultrasound-guided nutrition plan, (4) employing “body–mind” synchronous rehabilitation to facilitate functional recovery, (5) taking antirejection treatment and protective isolation measures, (6) monitoring and nursing thyroid function.

**Results:**

During the intensive care, the patient’s vital signs were stable. The patient was successfully weaned from the ventilator and transferred to the general ward for further treatment at 9 days postoperatively, and discharged upon recovery at 58 days postoperatively. The patient was in good condition during follow-up.

**Conclusion:**

This study provides reference for the care of patients who undergo similar transplantation in the future.

## 1. Introduction

Laryngeal carcinoma is a common head–neck malignancy, responsible for 1 to 5% of all tumor cases, and it is still mainly treated with surgery.^[1]^ Patients with extensive tumor invasion suffer dysphagia, loss of phonic function, and permanent incision of the trachea due to total laryngectomy. This seriously affects their quality of life. In 1998 and 2012, 2 successful cases of laryngeal transplantation were recorded worldwide. The patients’ senses of smell and taste were restored, while their swallowing and phonic functions normalized postoperatively. This implied an improved quality of life.^[2,3]^ Laryngeal transplantation is currently the best strategy for reconstructing the laryngeal function of patients and ameliorating their quality of life.^[4]^ However, it is still not popularized due to the following factors: donor scarcity, low survival rates of transplanted organs, difficulties associated with organ function reconstruction, rejection reactions, and ethical problems regarding laryngeal (as a non-vital organ) transplantation.^[5]^

In April 2023, the first case of successful laryngeal transplantation was reported in Asia.^[6]^ The patient underwent combined larynx–trachea–thyroid transplantation, and was discharged upon recovery at 40 days postoperatively. In July of the same year, “larynx–trachea–thyroid–hypopharynx–esophagus” multi-organ cluster transplantation was successfully performed in 1 case and for the first time. There is limited information available in literature regarding the postoperative care experience of these transplants. Following meticulous treatment and care by a multidisciplinary team, the patient was transferred from the intensive care unit (ICU) to the general ward at 10 days postoperatively. The patient’s wounds completely healed and he started drinking water and eating solid food at 18 days and 23 days postoperatively, respectively. The swallowing function recovered well, and all indicators were gradually corrected at 37 days postoperatively. The patient was successfully discharged at 58 days postoperatively, and was in good condition during follow-up. The key points and the patient’s experience in intensive care are subsequently reported.

## 2. Case presentation

### 2.1. Clinical information

#### 2.1.1. General information

A 52-year-old male patient was admitted to the hospital on July 8, 2023, with “hypopharyngeal malignant tumor” due to pain upon swallowing, foreign body sensation in the throat, coughing after drinking (for 7 months), and post-activity shortness of breath (for 6 months). The enhanced computed tomography (CT) scan showed a malignant tumor with cervical lymph node metastasis, and the patient was pathologically diagnosed with squamous cell carcinoma by neck mass biopsy. Due to significant shortness of breath, tracheotomy was performed on January 5, 2023. Two cycles of chemotherapy and immunotherapy were completed on January 29 and March 2. On April 27, an enhanced CT scan of the neck showed a soft tissue density mass shadow at the right portion of the epiglottis–glottis level plane, and the epiglottis, right vocal cord, and aryepiglottic folds were affected. The mass grew posteriorly toward the prevertebral space. It grew outwardly in close relation to the right common carotid artery, and upwardly, such that it appeared to be poorly demarcated from the upper esophagus, with uneven stenosis of the adjacent pharyngolaryngeal lumen. The right portion of the thyroid cartilage, cricoid cartilage, and bilateral arytenoid cartilages were also involved. Transplantation was considered as the best possible treatment option. The patient had a history of hypertension, diabetes mellitus, and coronary artery disease, and was under good control with oral medications.

#### 2.1.2. Surgical and intensive care procedures

On July 9, 2023, the patient underwent bilateral neck lymph node dissection, combined larynx–trachea–thyroid–hypopharynx–esophagus transplantation, and laryngeal function reconstruction. During the operation, the internal jugular vein was incised to resect the tumor and then repaired. The vein of laryngeal graft was bridged to the repaired internal jugular vein, while the cervical plexus and superior laryngeal nerves of the graft were reconstructed. After the operation, the patient was transferred to the transplantation ICU for protective isolation and treatment. He was fitted with various intubations such as the nasogastric tube, a tracheotomy tube, and 3 traumatic cavity drainage tubes in the neck. On the day of admission, the patient was given ventilator support. Management of analgesia and sedation was done following the doctors’ instructions. The Critical-Care Pain Observation Tool (CPOT) score was kept at 0 points and the Richmond Agitation-Sedation Scale (RASS) score was maintained at -2~-3 points. After undergoing intensive care for 9 days, the patient’s condition stabilized so he was transferred to the general ward for further treatment. Table 1 shows the detailed treatment.

**Table 1:** Detailed procedures of postoperative intensive care.

Time	Assessment of disease	Treatment measures
1 day postoperatively	(1) Thyroid ultrasound showed few blood flow signals in the left lobe of the thyroid gland and low arterial blood flow velocity. (2) Ultrasonic evaluation of gastrointestinal tract showed that the enteral nutrition indications were met..	(1) Circulation was stabilized; and attention was given to local skin color and temperature. (2) Patient was gavaged with 100 mL of 5% glucose injection via the nasogastric tube at 50 mL/h. (3) Multidisciplinary rehabilitation team was set up to start early rehabilitation exercises.
2 days postoperatively	Ratio of arterial partial pressure of oxygen to the fraction of inspired oxygen (PaO_2_/FiO_2_) was 431 mm Hg.	Weaned from the ventilator and received high-flow oxygen therapy, with FiO_2_ = 30%.
3 days postoperatively	Thyroid color Doppler ultrasound showed that bilateral thyroid blood flow had improved.	
6 days postoperatively	Drainage fluid from the neck wound cavity turned from light red to dark red, and the drainage volume was reduced to 0 mL.	Surgeon removed the 3 neck drainage tubes.
8 days postoperatively	Patient was in the delirious state, which manifested as acute changes in consciousness, attention disorders, and confusion.	Protective restraint, intensive analgesia and sedation management, as well as enhanced psychosocial support were provided.
9 days postoperatively	Patient was transferred to the general ward for further treatment.	

PaO_2_ = arterial oxygen partial pressure, FiO_2_ = fraction of inspiration O_2_.

### 2.2. Nursing intervention

Ensuring the survival of transplanted organs and promoting the organ functional reconstruction were the key objectives of postoperative care in the ICU. Afterward, rounds by the multidisciplinary team from the ICU, ENT & Head and Neck Surgery Department, Thyroid Surgery Department, Gastrointestinal Surgery Department, Organ Transplantation Center, Infectious Disease Center, Clinical Nutrition Department, Ultrasound Medicine Department, Mental Health Center and Rehabilitation Department were put in place. The treatment and care plans covering antisepsis and anti-inflammation, acid suppression and stomach protection, glucose control and anticoagulation, antirejection, nutritional support, and early rehabilitation were also formulated. All this was done to improve the survival chance of transplanted organs postoperatively and promote organ functional reconstruction.

#### 2.2.1. The survival chance of the transplanted organs improved by stabilizing the circulation and reducing anastomotic tension

##### 2.2.1.1. Blood supply to transplanted organs was guaranteed by stabilizing the circulation

It is reported that the incidence rate of early thrombosis following vascular anastomosis is about 15%.^[7]^ Thrombosis and decreased blood flow velocity caused by anastomotic stenosis and vascular torsion are the causes of insufficient organ blood supply, and transplantation failure.^[8]^ In this case, the organ donor had hyperthyroidism, and the organ recipient had a history of hypertension and diabetes. Hyperthyroidism and diabetes can lead to hypercoagulability. At 1 day postoperatively, the levels of antithrombin III and D-dimer were 69.6% and 0.88 mg/L FEU, respectively, and 0.4 mL of the nadroparin calcium injection was subcutaneously administered (quaque die). At 3 days postoperatively, the patient’s blood pressure fluctuated (115–159/47–86 mm Hg). As a result, the urapidil hydrochloride diluent was intravenously pumped to maintain the target blood pressure at 140/90 mm Hg and guarantee the effective blood supply to transplanted organs. During the drug therapy, the patient’s blood pressure remained stable, without any signs of bleeding. Ultrasound showed that there was no venous thrombosis and that the blood supply to transplanted organs was good.

##### 2.2.1.2. Strict position management was adopted to reduce anastomotic tension and promote wound healing

The patient was instructed to turn over along the axis early postoperatively to avoid neck torsion. The head of the bed was elevated to a 30° angle. A soft pillow was put under the patient’s head, thereby creating an angle of 15° to 30° between the bed surface and the head. This helped to maintain a neck anteflexion position. A soft pillow was placed under each thigh root and hip to maintain the position and reduce local skin pressure. With meticulous care, no anastomotic bleeding occurred. In fact, good wound healing was achieved.

#### 2.2.2. Goal-directed analgesia and sedation protocol, and preventing anastomotic fistula.

Insufficient anastomotic blood supply and excessive tension are the key factors that lead to postoperative anastomotic fistula,^[9]^ which causes thoracic and abdominal infection, results in transplantation failure and possible threats to the lives of the patients.^[10]^ Anastomotic tear which emanates from severe coughing could be avoided through accurate management of analgesia and sedation. Combined with gastrointestinal ultrasound, nutrition supply could also be monitored to avoid anastomotic fistula.

##### 2.2.2.1. Goal-directed management of analgesia and sedation

A goal-oriented strategy can enhance accurate management of analgesia and sedation, which effectively prevented delirium and improved immune stability and long-term prognosis On the same day of the operation, management of analgesia and sedation was done, with the CPOT score kept at 0 points and the RASS score at -2~-3 points.^[11]^ At 2 days postoperatively, the patient was successfully weaned from the ventilator. High-flow oxygen inhalation was provided at the site of tracheotomy. The analgesia and sedation states were adjusted to keep the CPOT and RASS scores at 0 and -1 point, respectively. At 8 days postoperatively, delirium was observed so protective restraint was put in place and management of analgesia and sedation were enhanced, after which the patient reverted to a calm state and cooperated with the treatment. The dosage was dynamically adjusted to keep the patient calm and relaxed, thereby preventing any adverse consequences that may be caused by improper management of analgesia and sedation.

##### 2.2.2.2. Airway care

Standardized airway management can effectively prevent infection and complications such as anastomotic fistula.^[9,12]^ Using the minimum closure technique, respiratory therapists titrated the cuff pressure to 20 cm H_2_O.^[12]^ The cuff pressure was monitored Q6h to avoid tracheal wall ischemia and compromised blood supply to transplanted organs. In the first 3 days postoperatively, bedside fiberoptic bronchoscopy was performed daily, while airway and pulmonary secretions were aspirated through the site of tracheotomy.

At 2 days postoperatively, the patient was weaned from the ventilator and received high-flow oxygen therapy. The cuff pressure was still maintained at 20 cm H_2_O because the swallowing function of the patient had not been established and the self-purification ability of the airway was poor. The major care measures for the airway were as follows: (1) The airways were warmed and humidified to enhance the normal physiological function of the respiratory mucosa; (2) compound ipratropium bromide solution and acetylcysteine solution were used for expectoration by aerosol inhalation; (3) sputum was aspirated as needed, with a vacuum pressure at 80 to 150 mm Hg (1 mm Hg = 0.133 kPa); (4) oral and nasal secretions were sucked using a flexible rubber hose at a depth not longer than the distance from the tip of the nose to the earlobe. Excessive vacuum pressure can cause mucosal bleeding and the anastomotic stoma can be damaged if the rubber hose goes too deep. Before sputum aspiration, fiberoptic bronchoscopy, positional changes, and other actions that might cause coughing, analgesics/sedatives or muscle relaxants were used to protect the airway.^[13]^ Therefore, the cough reflex was effectively prevented during the care procedures. Good airway anastomotic healing was achieved after standardized care.

##### 2.2.2.3. Esophageal care

The nasogastric tube was indwelt postoperatively for gastrointestinal decompression to reduce the risk of reflux, and enteral nutritional support. Due to postoperative anastomotic nonhealing and tissue edema, replacing the nasogastric tube may cause difficulties in intubation and anastomotic fistula. Therefore, the patient was informed of the importance of a nasogastric tube, which was doubly fixed to prevent unplanned extubation. No nasogastric tube displacement, high fever, chest pain, and dyspnea, were observed during the treatment and care. The signs and symptoms of anastomotic fistula, such as redness, tenderness, subcutaneous emphysema, and putrid pus outflow at the cervical wound skin, were also absent. The CT scan showed good anastomotic healing.

#### 2.2.3. The bedside ultrasound-guided nutrition plan was implemented

Early postoperative enteral and parenteral nutrition can facilitate gastrointestinal functional recovery, improve nutritional status, promote wound healing, and reduce the incidence rate of anastomotic fistula.^[14]^ The patient had a body mass index of 21.4 kg/m^2^, hemoglobin levels of 98 g/L, and albumin levels of 40.3 g/L. Therefore, the daily energy required was about 2000 kcal, including 100 g of protein. At 1 day postoperatively, fat emulsion (10%)/amino acid (15)/glucose (20%) injection was administered intravenously. To ensure the smooth implementation of the enteral nutrition plan and avoid gastric retention and aspiration, gastrointestinal evaluation was performed by nurses who specialized in ICU ultrasound. The enteral nutrition plan was formulated by nurses, doctors, and nutritionists. After the ultrasonic evaluation of the gastrointestinal tract revealed that enteral nutrition indications were met, enteral nutrition was initiated.^[15]^ At 7 days postoperatively, gavage with nutrient solution was suspended due to gastric retention, and total parenteral nutrition was provided until the patient was transferred to the general ward. Moreover, mosapride was gavaged and early rehabilitation exercise was undertaken to promote gastrointestinal motility. During enteral nutrition, no reflux or aspiration occurred and the nutritional status continued to improve. See Table 2 for the contents of the plan.

**Table 2:** Ultrasound-guided enteral nutrition plan.

Time	Results of ultrasound assessment	Details of enteral nutrition protocol	Patient’s symptoms
1 day postoperatively	Antral motility once every 2 min, antral motility index = 0.45, and intestinal motility.	Gavage with 100 mL of 5% glucose injection via the nasogastric tube at 50 mL/h, and with 5 mg of mosapride (Tid) to promote gastrointestinal motility.	No discomfort
2–6 days postoperatively	Antral motility index = 0.27–2.67, no gastric retention, and good intestinal motility.	Gavage with 100–200 mL of nutrient solution at 30–60 mL/h, Tid.	No discomfort
7 days postoperatively	Significant gastric retention; poor gastric contraction and dilatation.	Suspension of gavage with nutrition solution due to drainage of 260 mL of yellow-green gastric juice via the nasogastric tube.	Abdominal pain and distension.

Tid = ter in die.

#### 2.2.4. Functional recovery was promoted by “body–mind” synchronous rehabilitation

Factors such as loneliness caused by isolation treatment, as well as immobilization and ICU-acquired weakness that is induced by mechanical ventilation affect prognosis and increase the burden on individuals, families, and the society.^[16]^ To promote the recovery of patients’ physical, swallowing, and language functions, individualized early rehabilitation was initiated based on the 5P rehabilitation framework (Pain management and sedation, Position management, Physicotherapeutics, Pulmonary rehabilitation, and Psychosocial support). A nurse-led intensive rehabilitation care team was established, and through doctor–nurse–technician multidisciplinary cooperation, a rehabilitation plan was developed and dynamically adjusted according to the vital signs, pain scores, and muscle strength of the patient. The plan aimed to guide the neck–shoulder muscle exercise, relieve muscle stiffness, and improve swallowing and language functions. No adverse events such as rapid change in vital signs and accidental extubation occurred during the rehabilitation period. The swallowing function recovered well at 37 days postoperatively. The details of the rehabilitation plan are shown in Table 3.

**Table 3:** Details of the rehabilitation plan.

Time	Pain management and sedation	Position management	Physicotherapeutics	Pulmonary rehabilitation	Psychosocial support
1 day postoperatively	CPOT: 0 RASS: -3~-1	The head of the bed was elevated by 30°; patient assumed a neck anteflexion position	Grip training, ankle pump exercise, 30 min/time, Qd	Airway clearance by fiberoptic bronchoscopy, Qd; abdominal breathing training, 20 min/time, Bid	Sleep management (environmental optimization and drug assistance), and health education
2–4 days postoperatively	CPOT: 0 RASS: -2~-1	The head of the bed was elevated by >45°; patient assumed a neck anteflexion position	Upper limb lift training: (mainly shoulder and neck muscles), 6 times/group, 6 groups/time, Bid	Airway clearance by fiberoptic bronchoscopy, Qd; respiratory training, pulmonary re-expansion training, 20 min/time, Bid	Sleep management (environmental optimization and drug assistance), health education, encouragement of expression, and flexible visiting system
5–7 days postoperatively	CPOT: 0 RASS: -2~-1	The patient sat in bed, and the head of bed was elevated by >45°	Upper limb lift training: 6 times/group, 6 groups/time, Bid; bedside sitting position, 30 min/time, Qd	Airway clearance; respiratory training, pulmonary re-expansion training, 20 min/time, Bid	Sleep management, health education, encouragement of expression, and flexible visiting system
8 days postoperatively	CPOT: 0–4 RASS: -1~4	The head of the bed was elevated by 30–45°	Suspension of rehabilitation exercise due to delirium		Sleep management, health education, and family bedside accompany

*Note*: The patient was transferred to the ENT surgery ward at 9 days operatively. The bedside nurse was informed of the rehabilitation plan. Health guidance was given to the patient and his family, as the rehabilitation exercise continued.

CPOT = Critical-Care Pain Observation Tool, RASS = Richmond Agitation-Sedation Scale, Qd = quaque die.

The mental health assessment was done using the Huaxi Emotional-distress Index.^[17]^ The results showed that the patient had no mental health problems like depression. However, 12.7% of tumor patients face mental problems due to pain, fear of disease progression, and ignorance, so they need psychosocial support. At 8 days postoperatively, the patient developed delirium, so protective restraint was initiated, and intensive management of analgesia and sedation was implemented.^[18]^ The dosage of analgesics and sedatives was adjusted to relieve the patient’s physical discomfort; the relevance of transferring the patient to the ICU for protective isolation was explained to the patient; the family members were invited to accompany the patient at bedside; the recovery status of transplanted organs was explained to the patient by the ENT surgeon; and the patient was encouraged to communicate using a handwriting board or by typing on a mobile phone. The patient gradually recovered and cooperated with subsequent treatment.

#### 2.2.5. Immunosuppressive therapy and protective isolation measures were taken

Strictly follow the doctor’s advice on medication to maintain stable blood concentration, achieve the optimal immunosuppressive outcome, and reduce side effects.^[19]^ The patient is at high risk of infection so strengthened protective isolation and disinfection are necessary for avoiding infection.^[20]^ Disinfection measures were strictly put in place, and one-on-one care was given by nurses who specialized in transplantation. Tacrolimus capsules were administered at 8:00 and 20:00 every day, and the patient took mycophenolate mofetil tablets at 9:00 and 21:00 every day. Blood samples were collected between 6:00 and 6:30 every day to monitor the blood concentration. The blood concentration of tacrolimus was maintained at 10 to 12 ng/mL. The concentration of antirejection drugs was optimized to reduce possible side effects. During medication, no complications, rejection reactions, or catheter-related infections were noted.

#### 2.2.6. Monitoring and nursing the thyroid function

Hypothyroidism and hypocalcemia are common symptoms following total laryngectomy.^[21]^ At 1 to 6 days postoperatively, the blood gas analysis showed that the calcium ion concentration fluctuated between 1.050 and 1.110 mmol/L, so a calcium supplement was administered. At 3 days postoperatively, the levels of free triiodothyronine, free thyroxine, thyroid-stimulating hormone, reverse triiodothyronine, total triiodothyronine, and thyroglobulin were 24.60 pmol/L, 75.40 pmol/L, 0.183 mIU/L, 3.40 nmol/L, 5.64 nom/L, and >5000.00 μg/L, respectively. These findings suggested the existence of primary hyperthyroidism, possibly due to decreased thyroid function caused by immune rejection after transplantation. Under such circumstances, the care focused on the presence or absence of manifestations of hyperthyroidism, such as exophthalmos, palpitations, hyperhidrosis, finger trembling, and irritability. At 9 days postoperatively, the free triiodothyronine, free thyroxine, and thyroid-stimulating hormone levels were 10.60 pmol/L, 57.50 pmol/L, and 0.007 mIU/L, respectively. This implied that the thyroid function gradually recovered.

## 3. Limitations

This is the first report on the experience in nursing a patient receiving larynx-associated multi-organ cluster transplantation, and the findings can provide a reference for other similar surgical patients. Due to the short follow-up period of this case after discharge, less information is available about the survival of the transplanted organs and the patients’ quality of life. We will continue to regularly follow-up to acquire more information regarding the transplanted organs and the overall health of the patient.

## 4. Summary

The case highlighted herein was the first to report combined larynx–trachea–thyroid–hypopharynx–esophagus transplantation performed in our hospital. The key points of postoperative care were ensuring the survival of transplanted organs and promoting organ functional reconstruction later. During multi-organ transplantation, the functional status of the respiratory, digestive, and endocrine systems should be simultaneously given particular attention. Throughout the process, multidisciplinary cooperation is of paramount importance. The changes in the patient’s condition are dynamically monitored in real time to offer a basis for adjusting diagnosis and treatment plans. Moreover, individualized treatment nursing plans are formulated and implemented, thereby ameliorating the patient’s prognosis and quality of life.

## Author contributions

**Conceptualization:** Yaodan Zhang, Yu Xu, Aiping Du.

**Writing – original draft:** Jia Shen.

**Writing – review & editing:** Jia Shen, Huan Liu, Yongming Tian.
